# Research Progress on Polymer-Based Nanocarriers for Tumor-Targeted Delivery of Survivin siRNA

**DOI:** 10.3390/polym17172279

**Published:** 2025-08-23

**Authors:** Luya Ren, Shaoxia Wang, Bin-Chun Li, Guo-Bin Ding

**Affiliations:** 1 Institute of Biotechnology, Shanxi University, Taiyuan 030006, China; 2 Institutes of Biomedical Sciences, Inner Mongolia University, Hohhot 010070, China

**Keywords:** survivin siRNA, polymer-based nanocarriers, tumor-targeted delivery, cancer therapy

## Abstract

Survivin, a pivotal member of the inhibitor of apoptosis proteins (IAP) family, plays critical roles in cell cycle regulation and division. Survivin is overexpressed in most malignancies, making it an attractive therapeutic target. Due to its high specificity and potency, siRNA-based RNA interference (RNAi) has emerged as a powerful therapeutic strategy for effectively downregulating disease-related genes such as survivin in cancer therapy. However, naked siRNA suffers from rapid enzymatic degradation, poor cellular uptake, and off-target effects, severely limiting its therapeutic efficacy in vivo. Development of polymer-based nanocarriers for tumor-targeted delivery of survivin siRNA (siSurvivin) holds great potential to address these challenges. In this review, we first described the structure and function of survivin and summarized the survivin-targeted therapeutic strategy. Then, the siRNA delivery systems, particularly the polymeric nanocarriers, were introduced. Furthermore, a plethora of polymer-based nanocarriers for tumor-targeted siSurvivin delivery, including synthetic polymers (branched polymers, dendritic polymers, polymeric micelles), natural polymers (polysaccharides, proteins, and others), lipid-polymer hybrid nanoparticles, and polymer composite nanoparticles, were elaborated. Promising results underscore the potential of polymer-based nanocarriers for survivin siRNA delivery to enhance cancer therapy, providing a roadmap for future clinical translation.

## 1. Introduction

Cancer, as the second most common fatal disease in the world, has become a major public health problem that seriously threatens human health, with its incidence and mortality increasing year by year [1]. Its main feature is the uncontrolled growth of abnormal cells, which are able to break through the normal growth boundaries, invade the neighboring tissues, and even spread to other tissues of the human body through blood circulation and the lymphatic system [2]. Cancer, also known as a malignant tumor, is invasive and metastatic, and it is extremely destructive to the organism. The formation of cancer is usually associated with a variety of factors, including gene mutation, environmental factors, hereditary factors, and lifestyles [3]. These factors may trigger the activation of oncogenes or the inactivation of tumor suppressor genes, leading to abnormal cell proliferation. Traditional cancer treatments include chemotherapy, radiotherapy, and surgery. Although they can control cancer progression to a certain extent, the overall treatment effect is far from satisfactory due to their severe side effects [4].

In recent years, with the rapid development of nanotechnology, the application of nanoparticles in cancer therapy has gradually attracted extensive attention. Nanoparticles can leverage the enhanced permeability and retention (EPR) effect, then achieve passive targeted accumulation through the highly permeable blood vessels and damaged lymphatic drainage system of tumor tissues. To further enhance the accumulation of nanoparticles in tumors, antibodies, peptides, and other targeted ligands can be modified onto the surface of nanoparticles, enabling them to precisely recognize and bind to receptors on the surface of tumor cells [5]. Additionally, some studies have found that certain nanoparticles, even without modification or drug loading, can enhance anticancer effects by altering cell membrane permeability when used in combination with anticancer drugs [6]. This opens new possibilities for the application of nanotechnology in cancer therapy and provides innovative approaches for the delivery of nucleic acid drugs.

Small interfering RNA (siRNA), as a gene silencing tool, can specifically degrade target mRNA and inhibit the expression of specific genes [7]. Survivin, as a key member of the apoptosis inhibitory protein family, plays an important role in biological processes such as cell cycle regulation, cell division, and apoptosis inhibition, and exhibits overexpression in a variety of cancer cells [8]. Therefore, by designing siRNAs targeting the *survivin* gene, specific degradation of survivin mRNA can be achieved, and then the expression of *survivin* gene can be downregulated, thereby effectively inhibiting the proliferation of tumor cells. Based on this, targeted delivery of siRNA to silence the *survivin* gene in tumor cells has become a highly promising cancer treatment strategy.

Despite the great advantages of siRNA in cancer therapy, it still faces many challenges. First, siRNA has poor stability under physiological conditions and is easily degraded by nucleases, thus having a short half-life in blood [9]. Second, while siRNA exhibits high specificity, incomplete complementary pairing may occur within cells, leading to non-specific degradation and unpredictable changes in gene expression [10]. Additionally, siRNAs are difficult to enter into cells by themselves due to their anion charge [11]. These factors limit the efficacy of siRNA in cancer therapy. Therefore, developing carriers that can efficiently and safely deliver siRNA to target cells while enhancing its stability is crucial for fully realizing the therapeutic potential of siRNA. Polymeric nanocarriers are ideal delivery systems due to their biodegradability and biocompatibility [12]. They can form stable complexes with siRNA through electrostatic interactions or covalent bonding to protect siRNA from degradation [13]. However, some drawbacks of polymeric nanocarriers hindered their practical applications, such as low drug loading efficiency, high toxicity, uncontrollable drug release, and insufficient targeting. To address these issues, researchers have multifunctionally modified polymeric nanocarriers. For example, PEGylation of highly cytotoxic polyethyleneimine (PEI) significantly reduces its toxicity [14]; lipid modification not only reduces cytotoxicity but also further enhances transfection efficiency [15]. Additionally, by leveraging the unique physicochemical properties of the tumor microenvironment (TME), such as hypoxia, acidic pH, and elevated glutathione (GSH) levels, stimulus-responsive drug delivery systems can be designed to achieve targeted drug delivery and release [16]. Furthermore, combining lipids, inorganic materials, or metals with polymers to form composite delivery systems effectively overcomes the inherent limitations of polymers [17]. These innovative strategies not only optimize the drug-loading capacity and biocompatibility of nanoparticles but also significantly enhance siRNA delivery efficiency and therapeutic efficacy, providing promising solutions to overcome the limitations of traditional delivery systems.

**Figure 1:**
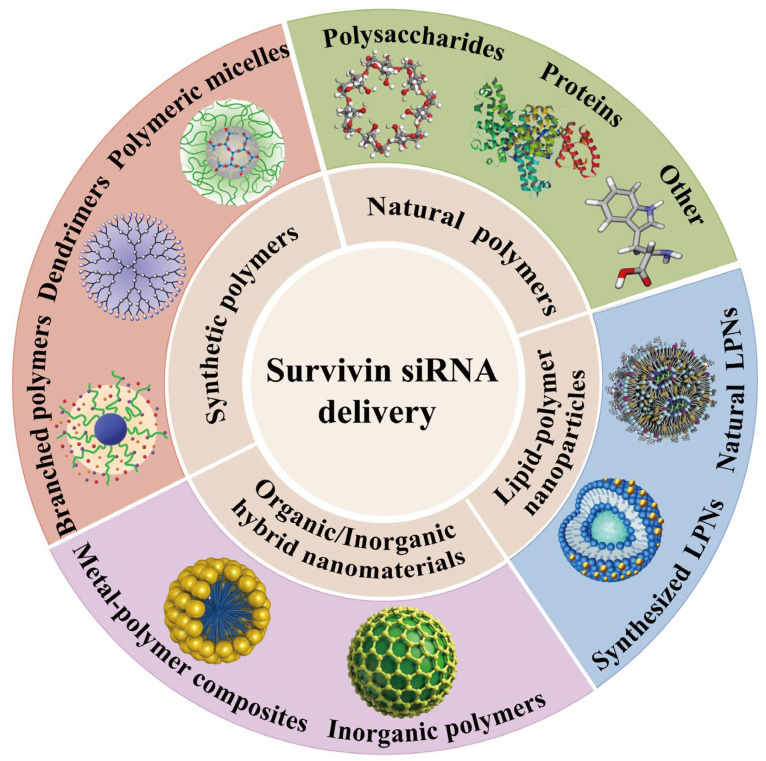
The classification of polymer-based nanocarriers for survivin siRNA delivery. There are mainly four kinds of polymeric nanocarriers for survivin siRNA delivery: synthetic polymers, natural polymers, lipid-polymer nanoparticles, and organic/inorganic hybrid nanomaterials.

In summary, an in-depth study of the mechanism of action of survivin, combined with advanced polymer nanocarrier-based delivery technology, will provide an important theoretical basis for the development of survivin-targeted siRNA delivery systems. This review stands out by systematically comparing various polymer nanoplatforms and their tumor-targeting strategies, offering unique perspectives on optimizing siRNA delivery efficiency. The aim is to provide an authoritative account and new insight into the research progress in recent years on the use of different polymer-based nano-platforms for tumor-targeted survivin siRNA delivery (Figure 1), thereby bridging the gap between preclinical studies and clinical applications in nanomedicine.

## 2. Introduction of Survivin

### 2.1. The IAP Family

The inhibitor of apoptosis proteins (IAPs) are a family of proteins and can directly bind to and inhibit caspases, effectively suppressing cell death by regulating cell division, cell cycle progression, and signal transduction. Meanwhile, IAPs are overexpressed in a variety of human cancers and preferentially expressed in malignant cells, so they are considered to be highly promising therapeutic targets, providing important directions and ideas for the design of cancer treatment strategies [18]. Currently, there are eight members of the IAP family, including NIAP, XIAP, cIAP1, cIAP2, ML-IAP, survivin, apollon, and ILP-2. Among them, XIAP, cIAP1, cIAP2, and survivin play important roles in tumor development, progression, and drug resistance due to their structural and functional uniqueness. Therefore, they have received extensive attention in recent years [19]. Survivin has emerged as a potential therapeutic target due to its key functions in cell cycle regulation, anti-apoptosis, promotion of angiogenesis, and metabolic reprogramming [20].

### 2.2. Survivin Structure

Survivin (BIRC5) is the smallest member of the Inhibitors of Apoptosis Proteins family, originally discovered by Ambrosini et al. in 1997, which is an evolutionarily conserved eukaryotic protein and exists as a stable homodimer [21]. Survivin consists of 142 amino acids and has a molecular weight of approximately 16.5 kDa. Compared to the structures of other members of the IAP family, survivin is relatively simple, containing only a BIR (Baculovirus IAP repeat) structural domain and an extended alpha helix. In addition, the first 10 amino acids at the N-terminus are proline-rich, representing the Mitochondrial Targeting Sequence (MTS) of survivin’s input to the mitochondria, and the α-helix region at the C-terminus has a Nuclear Export Signal (NES), which allows it to shuttle between the nucleus and the cytoplasm [21,22].

### 2.3. Survivin Function

**Figure 2:**
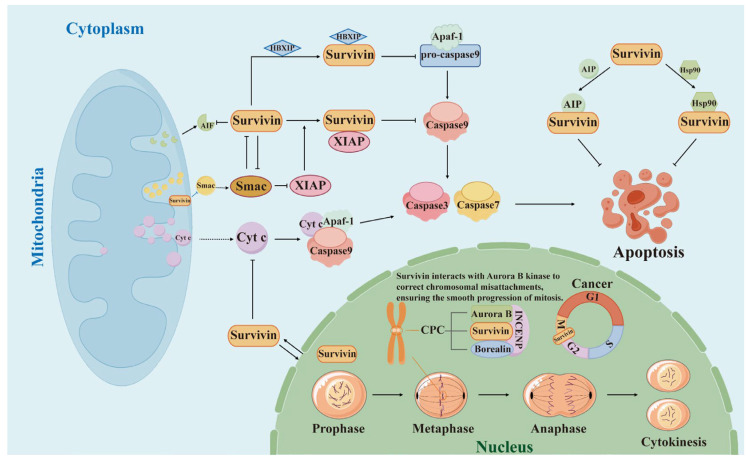
Schematic diagram of the function and mechanism of action of survivin. Survivin mainly inhibits apoptosis in the cytoplasm and mitochondria and regulates the cell cycle in the nucleus.

Survivin is widely distributed in the cell, mainly in the cytoplasm, and also prominently present in the nucleus and mitochondria [23]. Survivin exerts different biological functions in different organelles (Figure 2).

In the cytoplasm and mitochondria, survivin mainly exhibits apoptosis inhibition [22]. Unlike other members of the IAPs family, survivin does not directly bind to caspases but enhances the inhibitory effect on caspases by interacting with other IAPs family members (e.g., XIAP) to achieve anti-apoptotic effects. First, survivin is able to utilize the BIR structural domain to bind to another member of the X-linked inhibitor of apoptosis protein (XIAP) to form the survivin–XIAP complex, which enhances the inhibitory effect of XIAP on caspases [24]. XIAP, as a known inhibitor of caspases, can directly bind to caspase-3, caspase-7, and caspase-9 and thus inhibit their activities. By binding to XIAP, survivin enhances the anti-apoptotic function of XIAP, thereby indirectly inhibiting caspase activity [25]. In addition, the BIR structural domain of survivin can also bind to SMAC (Second Mitochondria-Derived Activator of Caspases), which exerts an anti-apoptotic effect [26]. SMAC/DIABLO is a mitochondria-derived pro-apoptotic protein, which is found mainly in mitochondria. And in normal cells, SMAC is localized in the mitochondrial membrane gap. When the cell is stimulated by apoptosis, mitochondria will release SMAC protein into the cytoplasm to bind with IAPs to abolish the inhibitory effect of IAPs on caspases, which promotes the activation of caspases and ultimately leads to apoptosis [27]. The binding of survivin to SMAC prevents SMAC from functioning properly, thus inhibiting the process of apoptosis and indirectly protecting IAPs from SMAC inhibition, maintaining the inhibitory effect of IAPs on caspases. Survivin also binds to HBXIP (HBx-Interacting Protein, Hepatitis B X Interacting Protein, a cofactor of survivin) and jointly inhibits apoptosis. The BIR structural domain of survivin interacts with HBXIP to form the HBXIP–survivin complex, which is capable of binding to pro-caspase-9, preventing the recruitment of pro-caspase-9 to apoptosis protease activating factor 1 (Apaf-1) [28]. Apaf-1 is a key regulatory protein in apoptosis, which activates caspase-9 and initiates the apoptotic cascade [29]. By blocking the binding of pro-caspase-9 to Apaf-1, the HBXIP–survivin complex inhibited the activation of caspase-9, thereby suppressing apoptosis. At the same time, survivin can also effectively prevent the release of cytochrome C from mitochondria to cytoplasm via regulating the Bax/Bcl-2 pathway, preventing the formation of the Cyt c/Apaf-1/Caspase-9 complex, and blocking the activation pathway of caspase-3, ultimately inhibiting the apoptosis [30]. In addition to effectively inhibiting caspase-dependent apoptosis, survivin also protects cells from apoptosis by preventing the release of AIF (Apoptosis-Inducing Factor) from mitochondria [31]. AIF, a protein located inside the mitochondria, plays an important role in regulating cell apoptosis. During the process of apoptosis, AIF is released from mitochondria and transferred to the nucleus, which in turn triggers chromatin condensation and DNA fragmentation and ultimately leads to apoptosis [32]. In addition, it has been reported that survivin binds to AIP [33] (aryl hydrocarbon receptor-interacting protein) and Hsp90 [22] (heat shock protein 90) and achieves anti-apoptotic effects via enhancing the stability of survivin.

In the nucleus, survivin is a key member of the chromosomal passenger complex (CPC) and plays an important role in cell cycle regulation, especially during mitosis. It is expressed in all phases of the cell cycle but reaches a peak in the G2/M phase and decreases significantly in the G1 phase [34]. CPC is a protein complex that plays a key regulatory role in mitosis and consists of four core components: aurora B kinase, INCENP, borealin, and survivin. Among them, aurora B kinase ensures the correct attachment of chromosomes to microtubules by phosphorylating kinetochore region proteins and activating the spindle assembly checkpoint (SAC). INCENP, survivin, and borealin are involved in the correct localization of the centromere [35]. Survivin interacts with aurora B kinase, regulates kinase activity, and assists it in correcting misconnections between chromosomes and spindle microtubules, and participates in the processes of chromosome separation, spindle assembly, and cytoplasmic division. This makes survivin a key regulator of cell mitosis and cell survival, assisting in the correct separation of chromosomes and ensuring that mitosis proceeds smoothly [36].

### 2.4. Survivin-Targeted Therapeutic Strategies

Survivin is typically expressed in developing and proliferating cells and is hardly expressed in normal cells. It is often overexpressed in most cancers, such as breast, liver, ovarian, lung, and prostate cancers, and is closely associated with the development, progression, and drug resistance of various cancers. Thus, survivin has received extensive attention as a potential target for tumor therapy [37]. Up to now, there are five surviving targeted cancer therapeutic strategies [38]. (a) Inhibitors that disrupt the interactions of survivin with its chaperone proteins. Examples include Shepherdin [39], AICAR [40] (disrupts the interaction between Hsp90 and survivin), PZ-6-QN [26], UC-112 and its analogs [41] (disrupts the interaction between survivin and SMAC). (b) Inhibitors that disrupt homodimerization of survivin. Examples include Abbot 8 [42], LQZ-7, LQZ-7i [43] etc. They degrade survivin by acting at the dimerization interface of survivin, thereby inhibiting cancer cell survival and promoting apoptosis. (c) Inhibitors that inhibit the transcription of the *survivin* gene. Inhibitors of *survivin* gene transcription include YM155, FL118 [44], and WM-127 [45], etc. Among them, YM155 is the first inhibitor targeting the survivin promoter, which can significantly inhibit survivin expression at the protein and mRNA levels and exert anticancer effects through a variety of mechanisms [46], but it has poor results in clinical trials and is mainly limited by stability issues [47]. FL118 and WM-127 also work by inhibiting *survivin* gene transcription to reduce its expression. (d) Survivin or its peptides for immunotherapy. This class mainly includes Survivin-2B80-88, SurVaxM (SVN53-67/M57-KLH), peptide cocktail vaccine EMD640744, and other specific survivin-based peptides, etc., which are mainly used for vaccine development and combination therapy and especially show great potential in combination therapy. (e) Inhibitors that induce survivin mRNA degradation. Inhibitors for the degradation of survivin mRNA mainly include antisense oligonucleotides (ASOs), ribozymes, and small interfering RNAs (siRNAs) [38]. Among them, two antisense oligonucleotides, ISIS 23722/LY2181308 and SPC3042/EZN-3042, are under investigation in clinical trials, but the results are not satisfactory [48]. Ribozyme-based treatment progressed slowly, and no significant result was obtained [49]. In contrast, siRNAs have been widely employed due to their high specificity, ability to achieve precise gene silencing through complete complementary pairing with target mRNAs, and simple design and synthesis at low cost [50]. However, the poor stability and low transfection efficiency of naked siRNAs greatly limited their application in tumor therapy [51], so delivery vectors are needed to improve their stability and cellular uptake efficiency.

## 3. siRNA Delivery Systems

### 3.1. Overview of siRNA Delivery Systems

Researchers have developed a variety of viral and non-viral vectors for siRNA delivery over the past years, aiming to enhance their stability, improve transfection efficiency, and reduce off-target effects. Viral vectors were developed relatively early, and these vectors have efficient transfection ability, and some lentiviral vectors and adeno-associated viral vectors are also capable of achieving long-term and stable expression of siRNAs [52]. However, the immunogenicity of viral vectors, the potential risk of viral recombination, and the biosafety issues associated with insertion mutagenesis, together with their complex preparation process, high production cost, and limited cargo capacity, have restricted their widespread use in clinical applications [53].

Due to the above-mentioned limitations of viral vectors, non-viral vectors are gradually gaining attention as an alternative. Non-viral vectors mainly include lipid nanoparticles [54], polymer nanoparticles [12], inorganic nanoparticles [55], and biomimetic nanoparticles [56], etc., which have the advantages of low immunogenicity, low off-target effects and toxicity, easy large-scale production, applicability to a variety of delivery routes, and long-term stability. In recent years, with the rapid development of nanotechnology and biomaterials science, the application of non-viral vectors in siRNA delivery has made significant progress, providing new possibilities for the clinical translation of siRNA agents. Among them, polymer-based nanoscale delivery systems have become a type of highly promising tool for siRNA, delivery especially in clinical applications, due to their excellent biodegradability, biocompatibility, and adjustable physicochemical properties [57].

### 3.2. Polymer-Based Nanoscale Delivery Systems

Polymer nanoparticles (PNPs) are prepared from natural or synthetic polymeric materials by physical or chemical methods, with the size usually ranging from 1 to 1000 nm [58]. Natural polymers such as hyaluronic acid, cyclodextrins, and chitosan exhibit significant advantages in drug delivery systems due to their unique physicochemical properties; for example, hyaluronic acid has good moisturizing and targeting properties and is able to specifically bind to the CD44 receptor, which enables precise drug delivery to tumor cells [59]. Due to its cationic properties, chitosan, is able to interact with the negative charge on the cell surface to enhance the cellular uptake and adhesion of drugs [60]. The unique molecular structure of cyclodextrins can be used to encapsulate drug molecules and improve drug stability and solubility [61].

Compared to natural polymers, synthetic polymeric composites have the characteristic of easy modification. Polymers such as polyethyleneimine (PEI), polypropyleneimine (PPI), polylactic acid (PLA), and polyethylene glycol (PEG) can be chemically modified to endow different drug release profiles. In addition, polymeric nanoparticles can achieve responsive drug release based on environmental factors (e.g., pH, temperature, enzymes, etc.) to further enhance therapeutic efficacy. This versatility and modifiability make polymer nanoparticles popular in the field of drug delivery [62].

However, with the increasing demands on the performance of conventional delivery vehicles and the limitations of liposomes (Lipid Nanoparticles, LNPs) in terms of stability and targeting ability, researchers began to explore some innovative solutions [63]. They combined lipids with polymers to develop lipid-polymer nanoparticles (LPNs), a hybrid structure that not only retains the biocompatibility of lipids but also enhances their stability by leveraging the properties of polymers. Meanwhile, the targeting ability and delivery efficiency of LPNs can be further enhanced by surface modification [64]. For example, polysaccharide-modified lipid nanoparticles showed higher stability and targeting ability in drug delivery [65]. Meanwhile, researchers have also attempted to combine metals [66] or inorganic materials [67] with polymers to further optimize delivery efficiency and reduce cytotoxicity. These innovative strategies provide new ideas and directions for the development of drug delivery systems.

Thus, we will systematically describe the research progress of polymer-based nanocarriers for targeted delivery of survivin siRNA from four aspects: synthetic polymeric nanoparticles, natural polymeric nanoparticles, lipid polymer nanoparticles, and other polymeric nanoparticles. The advantages and disadvantages of the four polymer-based survivin siRNA delivery systems were summarized in Table 1.

## 4. Polymeric Nanocarriers for Targeted Survivin siRNA Delivery

### 4.1. Targeted Delivery of Survivin siRNA by Synthetic Polymeric Nanoparticles

#### 4.1.1. Branched and Hyperbranched Polymers

Polyethyleneimine (PEI) is a highly cationic polymer, which is often regarded as the “gold standard” polymer in gene transfection due to its excellent transfection efficiency, gene complexation ability, and endosome escape activity [73]. However, high cytotoxicity limits its wide application [74]. To reduce cytotoxicity while maintaining efficient transfection, researchers have developed various modification strategies. In our previous study, polylactide (PLA) was integrated to polyethyleneimine via synthesizing PEI–PLA copolymer, PLA modification significantly improved the cytocompatibility of PEI and retained the high transfection efficiency [75] (Table 2). And pH low insertion peptide decoration further improved the biocompatibility of PEI–PLA copolymer, while pH-responsive and effective siRNA delivery was achieved [76].

Hamidreza et al. synthesized octanoic acid (OA)-, palmitic acid (PA)-, oleic acid (OA)-, and linoleic acid (LA)-modified PEIs by introducing lipids on low molecular weight PEIs (2 kDa). These lipid modifications significantly enhanced the cellular uptake efficiency and delivery capacity of PEIs, and reduced cytotoxicity. Among them, octanoic acid-modified PEI showed the best survivin silencing efficiency, which significantly reduced cell survival and induced apoptosis [77]. In 2018, Manoj et al. used linoleic acid-modified polyethyleneimine (PEI–LA) as the main carrier to deliver survivin siRNA and introduced polymers such as hyaluronic acid (HA), polyacrylic acid (PA), chondroitin sulfate (DS), and methylcellulose (MC) to optimize delivery efficiency and cellular uptake, which significantly improved cellular viability and cellular uptake when compared to PEI–LA. Compared with PEI–LA alone, their incorporation significantly improved the cellular uptake and release of siSurvivin, and, in particular,, the incorporation of HA and PA was able to effectively inhibit the growth of breast cancer cells with fewer side effects on non-malignant cells [78].

**Figure 3:**
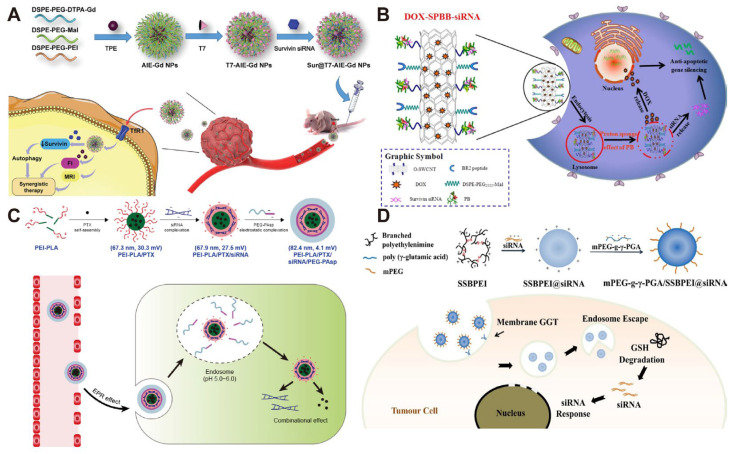
(**A**) The scheme of the fabrication and working principle of Sur@T7-AIE-Gd nanoparticles (NPs). Reprinted with permission from [79]. Copyright 2022, John Wiley and Sons; (**B**) Schematic diagram of DOX–SPBB–siRNA nanocarriers in A549 lung cancer cells. Reprinted with permission from [81]. Copyright 2019, American Chemical Society; (**C**) Schematical illustration of preparation of PTX/siRNA-loaded layer-by-layer nanoparticle delivery system and its intracellular therapeutic mechanism. Reprinted with permission from [82]. Copyright 2018, Dove Medical Press; (**D**) Schematic illustration of the targeted delivery of siRNAs to tumor cells by mPEG-g-γ-PGA/SSBPEI@siRNA nanoparticles. Reprinted with permission from [84]. Copyright 2020, Elsevier.

In addition to lipid modification, Hou et al. combined PEI with T7 peptide-modified AIE/Gd nanoparticles to form Sur@T7-AIE-Gd NPs, a nanocarrier with bifunctional imaging of magnetic resonance imaging (MRI) and aggregation-induced emission (AIE) imaging, which enabled precise delivery, real-time monitoring, and highly efficient treatment of hepatocellular carcinoma (HCC) (Figure 3A) [79]. In another pioneering study, Zhupanyn et al. for the first time combined natural extracellular vesicles (ECVs) derived from different cell lines with PEI to construct a complex for siRNA (siSurvivin) delivery. In a PC3 tumor xenograft nude mouse model, PEI/siSurvivin complexes modified with ECVs were injected into the tail vein and significantly inhibited tumor growth [80]. In addition, Cao et al. designed a pH-responsive polyethyleneimine betaine-functionalized single-walled carbon nanotube (SWCNT) complex for co-delivery of siSurvivin and DOX, and the results showed that the DOX-SPBB-siRNA complex significantly reduced the tumor volume in an A549 tumor xenograft nude mouse model (Figure 3B) [81]. In contrast, Jin et al. constructed a pH-responsive nanoparticle for co-delivery of paclitaxel (PTX) and siSurvivin by coating polyethyleneimine-poly (lactic acid) (PEI–PLA) with poly (ethylene glycol)-poly (aspartic acid) (PEG-PAsp) (Figure 3C). Due to the pH-responsive property of PEG-PAsp, the nanoparticles presented better drug release and cellular uptake at pH 5.5. In vitro experiments showed that the nanoparticles induced apoptosis in A549 cells (up to 80.81%) and blocked the cell cycle in the G2/M phase [82].

A high level of GSH in cancer cells is indispensable for reactive oxygen species (ROS) scavenging, which makes it a promising target for cancer therapy [90]. Wang et al. designed a multifunctional nanoscale system based on quantum dots (QDs)-modified hollow manganese dioxide (QH-MnO_2_) for the combined delivery of paclitaxel (PTX) and survivin siRNA to enhance chemotherapy efficacy. The delivery system first used SiO_2_ nanoparticles as templates, uniformly coating their surfaces with a layer of MnO_2_ via chemical vapor deposition to form SiO_2_@MnO_2_ composite structures. Subsequently, Na_2_CO_3_ solution was used to selectively dissolve the SiO_2_ templates. As the templates gradually dissolved, hollow MnO_2_ nanoparticles (H-MnO_2_) were ultimately obtained. Next, PTX is physically adsorbed onto the interior of H-MnO_2_, forming H-MnO_2_@PTX. Subsequently, InP/ZnS quantum dots are combined with H-MnO_2_@PTX via electrostatic interactions, forming QH-MnO_2_@PTX. To enable siRNA attachment, the surface of QH-MnO_2_@PTX is modified with polyethyleneimine (PEI) to impart a positive charge, enabling electrostatic interactions with siRNA to form QH-MnO_2_@PTX-siRNA. Following endocytosis, GSH-induced degradation of QH-MnO_2_ triggers the release of PTX and siRNA while restoring the fluorescence of InP/ZnS quantum dots, thereby enabling real-time tracking and precise localization of therapeutic drugs. This approach achieved a significant tumor inhibition rate of 95.3% in the MDA-MB-231 tumor-bearing nude mouse model [83].

Chen et al. designed a reduction-responsive nanocarrier (mPEG-g-γ-PGA/SSBPEI@siRNA) for effective siSurvivin delivery to A549 cells (Figure 3D). This nanocarrier consisted of a cationic carrier SSBPEI for siRNA complexation, an mPEG shell for nanocarrier stabilization, and γ-PGA for efficient cellular uptake [84]. The same group further designed dual-targeted and glutathione (GSH)-responsive nanoparticles (SSBPEI-DOX@siRNAs/iRGD-PEG-HA) for co-delivery of DOX and siRNA cocktails (survivin siRNA, Bcl-2 siRNA, and ABCG2 siRNA) to ovarian cancer stem cells. The system first connects BPEI-SH with DOX-SH through an oxidation reaction to form SSBPEI-DOX, followed by electrostatic interaction to complex with siRNAs, resulting in SSBPEI-DOX@siRNAs. To enhance the stability and targeting of the nanodelivery system, tumor-targeting peptide iRGD, hyaluronic acid (HA), and polyethylene glycol (PEG) were further incorporated, self-assembling via electrostatic interactions to form SSBPEI-DOX@siRNAs/iRGD-PEG-HA nanoparticles. The system targets the CD44 receptor on the membrane of CSCs via HA and the NRP-1 receptor via iRGD, enabling specific recognition and endocytosis of ovarian CSCs. Upon entering the cells, the disulfide bonds (-SS-) in SSBPEI-DOX can be specifically reduced due to the high glutathione content in the tumor microenvironment, thereby triggering the release of siRNAs and DOX. The experimental results showed that the nanoparticles significantly enhanced the antitumor effect of DOX compared with free DOX and also greatly suppressed the migration and invasion of A2780/DDP-derived CSCs [85].

These findings indicate that the cytotoxicity of PEI was significantly reduced and the anti-tumor effect was significantly enhanced by various modification strategies, providing ideas and methods for the application of PEI in tumor-targeted gene therapy.

#### 4.1.2. Dendrimers

Dendrimers are a class of highly structurally ordered synthetic macromolecules, rich in modifiable functional groups at the periphery of the molecule, which can significantly enhance the solubility of hydrophobic drugs, thereby enhancing their bioavailability and stability [91]. Common dendrimers include polyamide-amine (PAMAM) [92] and polypropyleneimine (PPI) [93]. In addition, cationic dendritic polymers can protect nucleic acids from degradation by binding to them through electrostatic interaction.

In this regard, Jugel et al. developed a PPI polymer-based siSurvivin-targeted delivery system, which achieved efficient cellular uptake and gene silencing of siRNAs through a caveolin/lipid raft-mediated internalization mechanism, thereby effectively inhibiting tumor growth. In this system, the PPI was modified with maltose, which significantly reduced cytotoxicity and dramatically improved biocompatibility; moreover, a single-chain antibody fragment (scFv) was introduced into the system, which was able to specifically recognize prostate stem cell antigens (PSCA), thus precisely entering into the target cells. Meanwhile, by taking advantage of the extremely high affinity between streptavidin and biotin, biotinylated scFv is stably bound to biotinylated PPIs by streptavidin, forming an intact polyplex structure and directing it to bind specifically to PSCA-positive cells. The luciferase activity assay indicated that the polyplex showed significant siRNA delivery efficiency and gene silencing effect in 293TPSCA/ffLuc cells. In the PC3PSCA xenograft tumor mouse model, PSCA-targeted siSurv polyplex reduced the tumor volume by about 50% compared to the control group, showing significant tumor growth inhibition [86]. Salve et al. designed a hyperbranched bis-MPA-based H_40_-TEPA-PEG-MUC1@siSurvivin delivery system. In this study, hyperbranched bis-MPA polyester (H_40_) was conjugated to tetraethylenepentamine (TEPA) to obtain H_40_-TEPA, and then H_40_-TEPA and mucin-1 (MUC1) aptamer were covalently coupled to the two ends of heterobifunctional polyethylene glycol (PEG) linker. The interaction of the MUC1 aptamer with the MUC1 receptor on MCF-cells greatly enhanced the cellular uptake of aptamer-conjugated targeted dendrimer. The experimental results showed that this delivery system exhibited a significant gene silencing effect in MCF-7 cells, which could reduce the expression level of Survivin mRNA by 2.5-fold compared with non-targeted dendritic polymers (1.3-fold) and Lipofectamine-2000 (2-fold), demonstrating a superior gene silencing efficiency [87].

#### 4.1.3. Polymeric Micelles

Polymeric micelles (PMs), as a class of highly efficient nanocarriers, have a unique “core–shell” structure that not only significantly improves the solubility, stability, and bioavailability of hydrophobic drugs [94], but also enables targeted delivery and stimulates responsive drug release through surface functionalization, thereby enhancing therapeutic efficacy and reducing systemic toxicity [95] (Table 2). Based on this property, Salzano et al. constructed a PM-based siRNA/PTX co-delivery system by conjugating siSurvivin to phospholipids (PL) via disulfide bonds and self-assembling with polyethylene glycol-phosphatidylethanolamine (PEG2000-PE) to form PM and by co-encapsulating paclitaxel (PTX). This system significantly inhibited cell viability (about 70% reduction in ovarian cancer cell line) and enhanced cell sensitivity to PTX in a variety of cancer cell lines, while effectively overcoming drug resistance by down-regulating survivin protein level and disrupting microtubule structure in drug-resistant cell line SKOV3-tr [88]. Subsequently, the same group further optimized the preparation process to increase the PTX encapsulation efficiency to 90% and verified its anti-tumor effect in the SKOV3-tr ovarian cancer drug-resistant nude mice model, where the survivin siRNA/PTX PM co-delivery system resulted in a 4-fold reduction in tumor volume compared to a single drug, as well as down-regulated the expression of survivin and promoted drug enrichment in tumor tissues [89].

### 4.2. Targeted Delivery of Survivin siRNA by Natural Polymeric Nanoparticles

#### 4.2.1. Polysaccharide

Chitosan (CS), a natural cationic polysaccharide, is the second most abundant polymer in nature after cellulose [96], and its surface amino groups can bind negatively charged substances (e.g., nucleic acids, proteins) by electrostatic interaction [97], a property that gives it a significant advantage in siRNA delivery systems as the only positively charged polysaccharide [98]. However, drug release issues and low solubility of chitosan at pH > 6.5 and in organic solvents limit its chemical modification and siRNA encapsulation efficiency [99]. Therefore, improving the solubility and drug release properties of chitosan is particularly important for the development of efficient siRNA delivery systems (Table 3).

**Figure 4:**
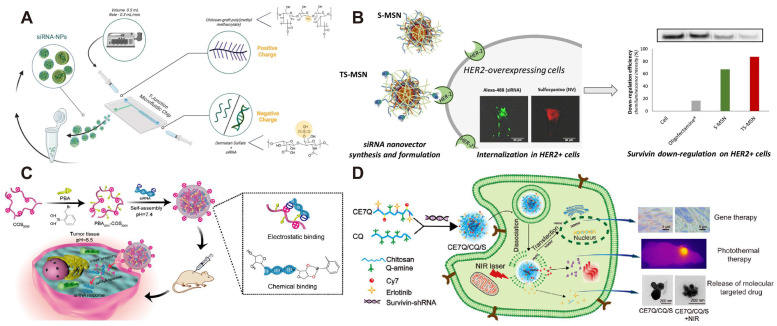
(**A**) The conceptual strategy to nanoencapsulate siRNA within doubly self-assembled CS/DS polymeric nanoparticles under a microfluidics setup. Reprinted with permission from [100]. Copyright 2025, Academic Press. (**B**) Synthesis of siRNA nanocarriers and internalization process in HER2+ cells. Reprinted with permission from [106]. Copyright 2019, Elsevier. (**C**) Schematic illustration for synthesizing PBA-modified COS. Reprinted with permission from [105]. Copyright 2021, Elsevier. (**D**) Near-infrared/pH dual-responsive nanocomplexes for targeted imaging and chemo/gene/photothermal tri-therapies of non-small cell lung cancer. Reprinted with permission from [101]. Copyright 2020, Elsevier.

It has been reported that chemical modifications (e.g., phosphorylation, carboxymethylation, quaternization, etc.) can significantly improve the solubility of chitosan and endow it with new functions [115]. Recently, Sader et al. constructed a dual self-assembled nanoparticle based on dermatan sulfate (DS) and chitosan, in which chitosan was used to form amphiphilic chitosan graft copolymer (CS-g-PMMA) by grafting poly(methylmethacrylate) (PMMA) and subsequently formed a polyelectrolyte complex (PEC) with DS via electrostatic interactions for the delivery of survivin siRNA (siSurvivin) (Figure 4A). Compared with the control group, 4T1 cells treated with siSurvivin-loaded nanoparticles showed a significant decrease in cell viability, migration ability, and sphere size, confirming that this delivery system is effective in achieving gene silencing [100]. Li et al. introduced quaternary ammonium into the hydroxyl groups at the C-6 position of chitosan via click chemistry to obtain 6-N, N, N-trimethyltriazole chitosan (CS) with high gene transfection efficiency. Erlotinib (Er), a near-infrared dye (Cy7), and quaternary ammonium were conjugated onto the chitosan skeleton to form CE7Q (Figure 4D). CQ and CE7Q were mixed and used to condense Survivin shRNA-expressing plasmid (SV) to obtain CE7Q/CQ/S nanocomplexes. This system could recognize epidermal growth factor receptor (EGFR) and enter into EGFR-mutated non-small cell lung cancer (NSCLC) cells, and a stimuli-responsive release profile was achieved by near-infrared laser irradiation at pH 5.4. CE7Q/CQ/S markedly downregulated survivin expression in all three cell lines (A549, PC9, and H1975) and displayed superior antitumor efficacy in vitro and in vivo by integrating chemo/gene/photothermal triple therapies into one nanoplatform [101]. On the other hand, Sun et al. successfully synthesized poly (ethylene glycol)-modified chitosan (PEG-CS) by coupling PEG molecules to the hydroxyl group of chitosan, and PEG-CS was employed to condense survivin siRNA to obtain PEG-CS/siRNA nanoparticles. PEG-CS/siSurvivin showed a significant inhibitory effect on 4T1 cells, with the cell survival rate decreasing from 62% to 48% and the apoptosis rate elevated from 5.32% to 16.03% as compared to the naked siRNA group. Furthermore, in a 4T1 tumor-bearing mouse model, PEG-CS/siSurvivin inhibited tumor growth and lung metastasis effectively [102].

In another study, Yang et al. directly grafted TAT peptides to the primary amino groups of chitosan (CS) to synthesize TAT-g-CS copolymer, which was used to bind survivin-targeted siRNA. The cellular uptake efficiency of TAT-g-CS/siRNA nanoparticles was 1.3-fold higher than that of unmodified CS/siRNA nanoparticles. TAT-g-CS/siRNA^Sur^ nanoparticles strongly inhibited the proliferation of 4T1-Luc tumor cells via triggering cell apoptosis and markedly suppressed the in vivo growth and metastasis of malignant breast tumors [103].

Construction of chitosan-based stimuli-responsive nanocarriers can significantly improve the drug release efficiency in tumor tissues, thus enhancing the therapeutic effect of siRNA delivery systems [116]. For example, Zhang et al. grafted p-mercaptobenzoic acid (MA) on *N, N, N*-trimethyl chitosan (TMC) to obtain MA-TMC (MT) polymer, which could self-assemble into MT nanoparticles (NPs) with a core–shell structure and redox-responsive property. Then, paclitaxel (PTX), survivin shRNA-expressing plasmid (iSur-pDNA), and recombinant human interleukin-2 (rhIL-2) were loaded into MT NPs to form MT/PTX/pDNA/rhIL-2 NPs. PTX was quickly released from MT/PTX/pDNA/rhIL-2 NPs under simulated intracellular reductive conditions (10 mM GSH), and the release rate reached up to 98% at 24 h, significantly higher than that of GSH-free conditions (31%). In addition, the tumor inhibition rate of MT/PTX/pDNA/rhIL-2 NPs reached 98.7% in the H22 tumor-bearing mice model, which was significantly higher than that of the treatment groups using the three drugs alone, indicating that this system could dramatically enhance the antitumor effect [104].

Due to the increased water solubility and non-toxicity as compared to chitosan, chitooligosaccharides (COSs, also known as chitosan oligomers or chitooligomers) received considerable attention for pharmacological and medical applications [117]. Phenylboronic acid (PBA)-modified COS (PBA-COS) was synthesized by Liu et al. and employed to deliver survivin-targeted siRNA (siSur) (Figure 4C). The prepared PBA_20%_-COS_3000_/siSur nanoparticles remarkably inhibited the cell proliferation and significantly inhibited the growth and distant metastasis of B16F10 melanoma cells in the melanoma mouse model [105].

David et al. developed a targeted stealth magnetic siRNA nanocarrier (TS-MSN) as a delivery system for delivery of survivin siRNA (Figure 4B) [106,107]. In this system, superparamagnetic iron oxide nanoparticles (SPIONs) was used as the core, while the shell was composed of polyethylene glycol (PEG), anti-HER2 single-chain antibody fragments (scFv), chitosan, poly-L-arginine (PLR), and survivin siRNA. TS-MSN showed an improved cellular internalization and enhanced the anticancer effect of DOX via downregulating survivin expression.

In addition to chitosan, amylose, as another natural polysaccharide polymer, has become an important carrier material in the field of drug delivery by virtue of its rich source of raw materials, good biocompatibility, and low toxicity [118]. Based on the unique properties of amylose, Zhang et al. developed CSP/TPE@siRNA-SP94 NPs for survivin siRNA delivery. Cationized amylose (CA), superparamagnetic iron oxide (SPIO) NPs, and tetraphenylethylene (TPE) self-assembled into nanospheres (CSP/TPE), followed by surface functionalization with S94 peptides and survivin siRNA loading. CSP/TPE@siRNA-SP94 NPs exhibited excellent in vitro and in vivo fluorescence and magnetic resonance imaging (MRI) properties and remarkably inhibited tumor growth in nude mice with Huh-7 tumors [108].

These innovative strategies not only effectively address the limitations of chitosan in drug delivery but also provide new research ideas and development directions for the application of chitosan and its derivatives in the biomedical field.

#### 4.2.2. Protein

Among the natural polymer systems, protein-based polymers (e.g., silk protein, fish protein, collagen, etc.) reduce the toxicity and immunogenicity of drugs by virtue of their excellent biocompatibility and biodegradability. Meanwhile, their rich functional groups can be coupled with drugs to realize precise drug delivery, showing significant application advantages [119] (Table 3).

Norouzi et al. prepared silk fibroin nanoparticles (SFNPs) by extracting silk fibroin protein from silkworms, then modified the surface of SFNPs with PEI through electrostatic interaction to enhance the binding capacity of the carrier with siRNA. Subsequently, by optimizing the ratio of SFNPs to DOX, they successfully constructed a DOX/siRNA/PEI/SFNPs nanodelivery system. This nanodelivery system had a significant antitumor effect in a 4T1 tumor-bearing mice model, with a tumor growth inhibition rate of 84.19%, and did not cause significant weight loss or other systemic toxicity, providing a new strategy for breast cancer treatment [109].

As a cationic peptide rich in arginine residues, protamine can bind to negatively charged nucleic acids to form stable complexes through electrostatic interactions, thus effectively protecting them from nuclease degradation [120]. Currently, the clinical safety of protamine has been verified and has shown excellent biocompatibility [106]. Based on the properties of protamine, Xu et al. developed an aptamer-protamine-siRNA nanoparticle (APR), which consists of protamine, ErbB3 aptamer, and siSurvivin, for targeting and recognizing ErbB3-positive breast cancer cells. In this system, the ErbB3 aptamer can specifically recognize and bind to the ErbB3 receptor overexpressed on the surface of breast cancer cells, realizing the precise delivery of siRNA. Meanwhile, protamine not only enhances the stability of the complex but also improves the intracellular delivery efficiency of siRNA through the electrostatic binding of siSurvivin. The apoptosis rate of cells is significantly increased to 37.90% after APR treatment as compared to 4.15% of the control group. APR nanoparticles demonstrated a significant therapeutic effect in the MCF-7-bearing nude mice model [110]. In another study, Ma et al. further improved the specific enrichment of the nanocarriers in tumor tissues by loading both siSurvivin and Pt (IV) prodrugs using protamine/hyaluronic acid nanocarriers, Pt (IV) was integrated into the nanoparticles through chemical conjugation with the coating polymer polyglutamic acid (PGA). Surface modification of the nanocarriers by PGA effectively enhanced their stability and prolonged the circulation time in blood. In a nude mouse A549/DDP tumor model, this delivery system achieved 82.46% tumor growth inhibition [111].

#### 4.2.3. Others

In addition to polysaccharides and protein-based natural polymers, other natural materials have been widely used in the design of delivery systems [121] (Table 3). For example, Liu et al. conjugated the carboxyl group of lysine (-COOH) with hydrazine (-NH-NH_2_) to form lysine-hydrazine, which was subsequently polymerized with HG-CA monomer under acidic conditions (pH 5.0) to form a biodynamic polymer (Lys-biodynamer) with dynamic covalent bonds. This polymer is pH-responsive, capable of releasing siSurvivin in acidic environments, has good biocompatibility and low cytotoxicity, and has dynamic covalent bonds such as imine and acyl-hydrazone bonds in its structure that can be reversibly broken and reorganized in specific environments. The adaptability of the polymer in different environments enables it to remain stable in complex biological environments while releasing the drug when needed to achieve controlled release [112]. Han et al. linked phenylalanine dipeptide (FF) with PEG–Arg–Ala (PEG–RA) sequence at the N-terminus to improve its hydrophilicity and serum stability. Arginine is frequently present in cell-penetrating peptides, which can enhance cellular uptake. Alanine serves as a spacer, which effectively avoids the steric hindrance [113].

Zhao et al. designed VE-Su-Sper/DSPE-PEG_2000_/siSurvivin nanocomplexes by taking advantage of the properties of spermine and vitamin E. Spermine is able to specifically interact with the major groove of the siRNA helix, while vitamin E enabled the complexes to interact with the structural domains of lipid rafts on the cellular membranes, which effectively avoided lysosomal trapping and improved the intracellular delivery efficiency of siRNA. This nanocomplex was able to effectively silence the Eg5 and Survivin genes and arrest the cell cycle at the G2/M phase. VE-Su-Sper/DSPE-PEG2000/siSurvivin nanocomplex significantly inhibited the tumor growth in HepG2-tumor-bearing mice, and the tumor volume and weight were about 4-fold smaller than other groups, and survivin expression was significantly reduced in tumor tissues [114].

### 4.3. Targeted Delivery of Survivin siRNA by Liposomal Polymer Nanoparticles

Lipid polymer hybrid nanoparticles (LPHNPs) are hybrid delivery systems with a polymeric core enclosed by a lipidic shell [122]. Integrating the structural properties of liposomes and polymers. The polymeric core is commonly composed of polylactic acid (PLA), polycaprolactone (PCL), pluronic F-68, chitosan, etc., while the lipid shell is made up of myristic acid, phosphatidylcholine (PC), cholesterol, 1,2-dipalmitoylsn-glycero-3-phosphocholine (DPPC), stearic acid, 1,2-distearoyl-sn-glycero-3-phosphoethanolamine (DSPE), soya phosphatidylcholine (SPC), and 1,2-dilauroyl-sn-glycero-3-phosphocholine (DLPC). LPHNPs combine the good structural integrity, storage stability, and controlled release quality of polymeric cores with the great biocompatibility and bioavailability of lipid layers [71].

**Figure 5:**
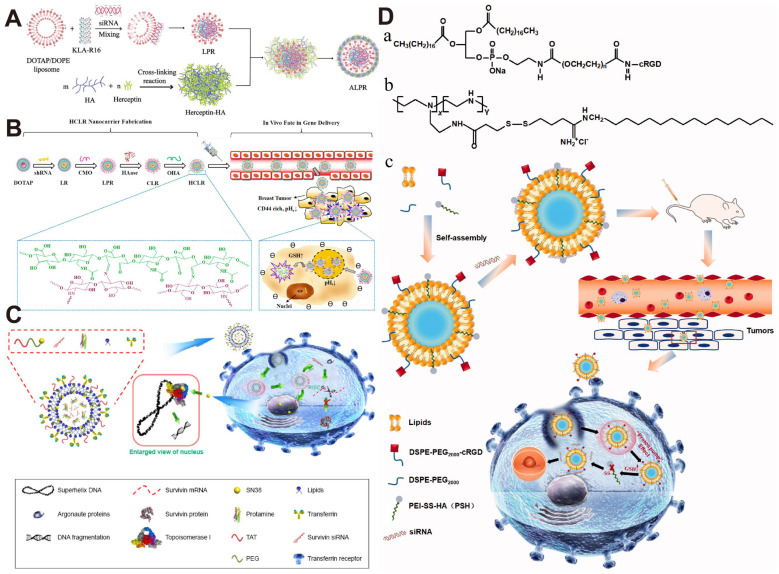
(**A**) Schematic illustration of the preparation of ALPR by embedding LPR into Herceptin-HA. Reprinted with permission from [125]. Copyright 2023, Wiley-VCH Verlag; (**B**) Schematic of HCLR Nanocarrier Fabrication and the In Vivo Fate in Breast Tumor Targeting Gene Delivery. Reprinted with permission from [130]. Copyright 2020, American Chemical Society; (**C**) The liposome structure and effect mechanisms of Tf-L-SN38/P/siRNA. Reprinted with permission from [127]. Copyright 2018, Dove Medical Press; (**D**) The structural formulae of DSPE-PEG 2000-cRGD, PSH, and schematic diagram of the structure of cRGD-PSH-NP and its application for siRNA delivery to cancer cells. (**a**) Structural formulae of DSPE-PEG2000-cRGD. (**b**) Structural formulae of PSH. (**c**) Schematic diagram of the structure of cRGD-PSH-NP and its application for siRNA delivery to cancer cells. Reprinted with permission from [126]. Copyright 2021, Taylor and Francis.

CD44 is a non-kinase transmembrane glycoprotein, and its high expression is associated with enhanced migration and invasion of tumor cells and resistance to chemotherapy [123]. Overexpression of HER2, a tyrosine kinase transmembrane glycoprotein, can lead to abnormal proliferation and enhanced invasiveness of tumor cells in breast cancer patients, which accelerates the tumor progression and leads to poor prognosis [124]. Notably, recurrent breast cancers may show CD44 and HER2 co-expression or single expression patterns. Therefore, dual-targeting strategies against CD44 and HER2 can more comprehensively cover different subtypes of tumor cells and improve the precision and effectiveness of treatment. Chen et al. developed a HER2/CD44-targeted hydrogel nanobot (termed as ALPR) by embedding the cationic liposome DOTAP/DOPE-based nanocomplexes containing pro-apoptotic peptide and survivin siRNA into Herceptin/hyaluronic acid cross-linked nanohydrogels (Herceptin-HA) (Figure 5A). ALPR can efficiently deliver Herceptin, peptide, and survivin siRNA to SKBR-3, MDA-MB-231, and MCF-7 cells. Moreover, ALPR exhibited excellent antitumor effects on SKBR-3 and MDA-MB-231 heterogeneous xenograft models with a tumor growth inhibition higher than 93% [125].

Yang et al. developed an siRNA delivery system, cRGD-PSH-NP, based on a modified polyethyleneimine (PSH) and DSPE-PEG2000-cRGD (Figure 5D). cRGD-PSH-NP loaded with survivin siRNA (cRGD-PSH-NP/S) was made up of egg phosphatidylcholine, cationic PSH, PEGylated lipids, survivin siRNA, and a targeting ligand (cRGD peptide). cRGD-PSH-NP/S markedly downregulated survivin expression both in vitro and in vivo and exhibited potent tumor inhibition (74.71%) in HepG2-bearing nude mice [126]. Bi et al. designed a transferrin (Tf)-functionalized and liposome-based system (Tf-L-SN38/P/siRNA) for codelivery of SN38 prodrug (a topoisomerase inhibitor) and survivin siRNA (Figure 5C). Tf-L-SN38/P/siRNA was more effective than liposomes carrying a single component and induced potent tumor inhibition (76.8%) in HeLa cell xenograft tumor-bearing nude mice [127]. Gibson prepared switchable lipid nanoparticles (switchable LNP) using CSL, DSPC, cholesterol, and DSPE-PEG2000 by microfluidics and manual extrusion methods, and LNP was used for survivin siRNA complexation to obtain siLNP. They found that survivin downregulation by siLNP enhanced the cytotoxicity of carboplatin and melphalan to Y79 and primary retinoblastoma cells [128].

In addition to being modified with synthetic polymers, liposomes can also be decorated with polysaccharides such as chitosan [129]. Chen et al. constructed a stimuli-responsive polysaccharide-enveloped liposome by layer-by-layer deposition of chitosan (CS) and hyaluronic acid (HA) onto the liposome and then loading survivin-shRNA gene and hyaluronidase (HAase) sequentially (Figure 5B). The as-prepared HA/HAase/CS/liposome/shRNA (HCLR) In the HA/HAase/CS/liposome/survivin-shRNA (HCLR) system, HA promoted tumor targeting by virtue of its specific binding to the CD44 receptor on the surface of tumor cells, resulting in much higher tumor accumulation than unmodified liposomes, and prolonging the retention time of liposomes in the blood circulation due to its negative charge. CS undergoes protonation in the tumor microenvironment (pH 6.5) to promote cellular uptake, resulting in a cellular uptake higher than 85%, which was far greater than pH 7.4. Meanwhile, pH-triggered HAase release decreased the extracellular matrix density and improved the diffusion of nanocarriers into tumor tissues, accounting for the better tumor penetration ability of HCLR. HCLR exhibited superior in vivo tumor suppression capability via downregulation of survivin and triggering cell apoptosis, and no visible damage was observed in normal tissues [130]. Similarly, Eljack’s team designed lipid-based nanoparticles (LBNP) for the co-delivery of tyrosine kinase inhibitor (TKI) Lapatinib (LAPA) and survivin siRNA (siSurvivin). This nanocarrier is based on lipid nanocapsules (LNCs) coated with a cationic chitosan shell. Chitosan was grafted on the surface of LNCs via a transacylation reaction. The siSurvivin-LAPA_LBNP had favorable physicochemical properties, showed a high cellular uptake in human epidermal growth factor receptor 2 overexpressed (HER2+) breast cancer cell line SK-BR-3, and exerted a significant synergistic cytotoxic effect as compared to siCtrl.-LAPA_LBNP. The IC_50_ value of siSurvivin-LAPA_LBNP to SK-BR-3 cells was 76.8 nM, which is much lower than LAPA ditosylate (159.0 nM) and siCtrl.-LAPA_LBNP (99.7 nM) [131]. In addition, Xie et al. developed a one-pot modular assembly strategy by combining octreotide, a cell-penetrating peptide (CPP), and glutamic acid. The three modules were assembled on the surface of the siRNA/liposome complex to obtain a multifunctional integrated survivin siRNA delivery system (OCA-CC). OCA-CC demonstrated enhanced cytosolic delivery in three tumor cells (A549, MCF-7 and PANC-1) and displayed remarkable antitumor efficacy in vitro and in vivo via silencing survivin expression [132].

It can be realized that the design of lipid-polymer hybrids breaks through the limitations of the traditional delivery system and significantly improves the targeting and cellular uptake efficiency of the drug delivery systems, which provides a new strategy for the design of future drug delivery systems (Table 4).

### 4.4. Targeted Delivery of Survivin siRNA by Organic/Inorganic Hybrid Nanomaterials

Nanohybrids integrate the biocompatibility of organic polymers/peptides and diverse functionalities of inorganic nanomaterials, and these nanohybrids have been widely employed in various biomedical applications, including drug delivery and cancer immunotherapy [17]. Calcium phosphate (CaP) nanoparticles have been developed for DNA and siRNA delivery due to the simplicity of preparation, intrinsic biocompatibility, and biodegradability [133]. For example, the CaP nanoparticle-based siSurvivin delivery system designed by Mitrach et al. achieved highly efficient loading of siRNA through electrostatic binding and acidic environment-triggered siRNA release. In addition, the introduction of a new terpolymer (o14PEGMA) also improved the dispersion stability of the nanoparticles, enhanced the cellular uptake efficiency, and significantly improved the delivery performance of the vector while reducing the potential cytotoxicity of the conventional CaP [134]. Kara et al. modified the CaP nanoparticles with arginine to simultaneously bind two siRNAs against survivin and cyclin B1. CaP-Arg-siRNAs significantly suppressed the expression of survivin and cyclin B1, resulting in a marked decrease in cell growth and induced apoptosis drastically [135]. Bi et al. synthesized RGDV-functionalized nanodiamond NDCONH(CH_2_)_2_NH-VDGR for survivin siRNA delivery. The NDCONH(CH_2_)_2_NH-VDGR/survivin-siRNA nanoparticle reduced survivin expression and displayed a potent inhibitory effect on tumor growth in vitro and in vivo [136].

Carbon nanotubes (CNTs), a nanoscale tubular structured material formed by carbon atoms arranged in a hexagonal pattern, have been widely used in biomedical applications due to their excellent thermal and optical properties. CNTs are able to efficiently convert near-infrared light into heat energy for photothermal therapy (PTT) and can be utilized to efficiently load different drugs in a covalent or non-covalent manner [137]. However, CNTs still face many challenges in biomedical applications, such as their inherent cytotoxicity and immunoreactivity as well as their dispersion in physiological environments, which limit their applications to some extent [138]. Therefore, to overcome these challenges and further enhance the potential of CNTs in biomedical applications, Zhao’s team introduced temperature-sensitive lipid peptide lipid (PL) and sucrose laurate (SL) to modify the single-walled carbon nanotubes (SCNTs) and multi-walled carbon nanotubes (MCNTs) for the delivery of survivin siRNA, and the modified CNTs demonstrated excellent biocompatibility and temperature-sensitive properties. Agarose gel electrophoresis showed that siRNA release rates from SCNT-PS/siRNA and MCNT-PS/siRNA were 80% and 67%, respectively, between 40 and 43 °C. The modified CNTs (SCNT-PS and MCNT-PS) did not show significant cytotoxicity to both HeLa cells and MCR-5 cells at high concentrations and had good cytocompatibility. Meanwhile, the combination of photothermal therapy (PTT) and gene therapy (GT) triggered substantial apoptosis in HeLa cells, with apoptosis rates of 63.8% and 60.2% for the SCNT-PS and the MCNT-PS groups, respectively. More importantly, SCNT-PS/siRNA and MCNT-PS/siRNA combined with PTT exhibited strong in vivo antitumor activity in nude mice with HeLa tumor xenografts, and the tumors disappeared completely in two mice in the SCNT-PS+P+G group [139].

Nanoscale metal–organic frameworks (MOFs) have shown high radio-enhancement effects by a unique radiotherapy-radiodynamic therapy mechanism. Ma et al. developed a hafnium-based cationic metal–organic layer (CMOL) for the delivery of siRNA cocktails targeting survivin, HIF-1α, and TGF-β to enhance the effect of cancer radiotherapy and to overcome the radioresistance of tumor cells. In this system, CMOL functions as an effective reactive oxygen species (ROS) generator, and the cationic framework serves as an siRNA cocktail carrier to synergistically downregulate survivin levels. It was shown that the cellular uptake of CMOL@ siRNA in 4T1 cells increased by almost 10-fold as compared to free siRNA. In combination with low-dose X-ray irradiation, CMOL@ siRNA exerted remarkable antitumor efficacy with 96.9% and 91.4% tumor growth inhibition in subcutaneous CT26 and 4T1 tumor models, respectively [140]. Cai et al. designed and prepared a cell-penetrating peptide (CPP)-functionalized metal–organic framework nanoplatform (termed PEG-CPP33@NPs) for co-delivery of oridonin (ORI) and survivin siRNA. The results showed that PEG-CPP33@NPs significantly increased the cellular uptake efficiency of siRNA in A549 cells and showed stronger tumor accumulation as compared with unmodified ZIF-90. In addition, PEG-CPP33@NPs induced a much higher apoptosis rate (60.73%) in A549 cells than unmodified siRNA@ZIF-90 (30.13%) and displayed the strongest in vivo antitumor effect with a tumor inhibition rate of 61.04% via co-delivery of ORI and survivin siRNA [141]. Additionally, Wan et al. achieved specific recognition and binding of the nanocarrier to the asialoglycoprotein receptor (ASGPR) on the surface of hepatocellular carcinoma (HCC) cells by modifying the surface of the metal–organic framework (MOF) with N-acetylgalactosamine (GalNAc), thereby enabling efficient delivery of survivin siRNA. Both in vitro and in vivo experimental results demonstrated that GalNAc-modified MOFs could specifically accumulate in HCC tumor tissues and were efficiently internalized by HCC cells. The protective effect of MOFs effectively enhanced the stability of siRNA, significantly downregulating survivin expression in HCC tumors, thereby inhibiting cell proliferation and inducing apoptosis to achieve tumor growth suppression [142].

All these studies have shown that the combination of inorganic materials/metal frameworks and polymers not only bridges the gap between the two but also significantly improves the overall performance of the nano-delivery system, which provides an effective strategy for overcoming radioresistance and enhancing the efficacy of radiotherapy (Table 5).

## 5. Conclusions

In summary, the survivin siRNA delivery system based on various polymers effectively overcomes the limitations of siRNA, such as poor stability, easy degradation, immunogenicity, and low delivery efficiency. By constructing various polymer-based nano-delivery systems, such as the synthetic polymer nano-delivery system with PEI as the core, the natural polymer nano-delivery system with chitosan as the core, and the composite delivery system combining lipids/inorganic materials/metals and polymers as introduced in this paper, not only did they improve the stability and delivery efficiency of siRNAs, but they also reduced the cytotoxicity of the delivery system, which made the survivin siRNA delivery system more effective. Survivin siRNA can exert its antitumor effect more effectively and inhibit the proliferation, invasion, and drug resistance of tumor cells, providing new strategies and ideas for cancer treatment. Meanwhile, the multi-drug combination therapy strategy shows a broad prospect in the field of tumor treatment. Combining the survivin siRNA-based gene therapy with various therapeutic approaches, such as chemotherapy, radiotherapy, immunotherapy, and multimodal imaging technology, it is expected to overcome the limitations of single therapy and yield new breakthroughs and hope for cancer treatment.

## Figures and Tables

**Table 1:** Advantages and disadvantages of four nanoparticle delivery systems.

Delivery System	Advantages	Disadvantages	Ref.
Synthetic polymers	Ease of functionalization, high stability, high drug loading capacity, and long circulation time enable sustained drug release.	Certain toxicity, immunogenicity, and safety risks; the preparation process is complex.	[68]
Natural polymers	Wide range of sources, good biocompatibility and biodegradability, low toxicity, and non-immunogenicity.	Off-target effect, stability, and longevity during storage and circulation in complex biological environments	[69,70]
Lipid-polymer nanoparticles	Stability, high loading of cargo, increased biocompatibility, rate-limiting controlled release, and prolonged drug half-life.	High dose may cause immune reactions, preparation is complex and costly, and long-term stability still needs to be studied.	[71]
Organic/inorganic hybrid nanomaterials	Combining the high stability and targeting properties of inorganic materials with the biocompatibility and biodegradability of organic materials, this approach enhances the intelligent response capability and structural stability of the carrier. Easy functionalization and low cytotoxicity.	Complex design, high preparation costs, batch-to-batch consistency issues, scalability for large-scale production, and biosafety.	[72]

**Table 2:** Targeted delivery of survivin siRNA by synthetic polymeric nanoparticles.

Synthetic Polymer-Based Survivin siRNA Delivery Systems					
Nanoparticle	Survivin Sequence	Cancer Types	Cell Lines	Outcome	Ref.
PEI-CA6.9/siRNA	-	Breast cancer	MDA-MB-231 cell	Octanoic acid-modified PEI showed the best survivin silencing efficiency, which significantly reduced cell survival and induced apoptosis.	[77]
CDC20/surviving siRNA polymer polyplexes	-	Breast cancer	MDA-MB-231 MDA-MB-436 MCF-7 cell	Polyethyleneimine modified with linoleic acid (PEI–LA) significantly increased cell viability and cellular uptake, improved the cellular uptake and release of siSurvivin, and effectively inhibited the growth of breast cancer cells.	[78]
Sur@T7-AIF-GD NPS	-	Hepatocellular carcinoma	LM3 cell	T7 peptide enhances targeting, dual-functional imaging with Gd and AIE magnetic resonance imaging (MRI) and aggregation-induced emission (AIE) imaging, enabling precise delivery, real-time monitoring, and efficient treatment of hepatocellular carcinoma.	[79]
ECV-modified PEI/siRNA	Sense: 5′-AUUCACCAAGGG-UUAAUUCdTdT-3′	Prostate and Osteosarcoma carcinoma Ovarian and Colorectal cancer	SKOV3 PC3 HCT-116 Saos-2 cell	In the PC3 tumor xenograft nude mouse model, the ECV-modified PEI/siSurvivin complex significantly inhibited tumor growth.	[80]
DOX-SPBB-siRNA	Sense: 5′-GAGCAGUUUGAAGAAUUATT-3′ Antisense: 5′-UAAUUCUUC-AAACUGCUUCTT-3′	Lung cancer	A549 cell	DOX-SPBB-siRNA complex significantly reduced the tumor volume in an A549 tumor xenograft nude mouse model.	[81]
PEI–PLA/PTX/siRNA-/PEG-PAsp	Sense: 5′-GCAUUCGUCCGG-UUGCGCUTT-3′	Lung cancer	cell	PEG-PAsp has pH-responsive properties, enabling the PEG-PAsp-modified delivery system to induce apoptosis and arrest the cell cycle at the G2/M phase in A549 cells.	[82]
QH-MnO_2_@PTX-siRNA	Sense: 5′-CACCGCAUCUCU-ACAUUCATT-3′ Antisense: 5′-UGAAUGUAG-AGAGCGGUGTT-3′	Breast cancer	MDA-MB-231 cell	MnO_2_ nanoparticles exhibit superior responsiveness to high levels of GSH within cancer cells. The fluorescence recovery function of InP/ZnS quantum dots enables real-time monitoring of drug release in the tumor microenvironment.	[83]
mPEG-g-γ-PGA/SSBPEI@siRNA	-	Lung cancer	A549 cell	PEI containing disulfide bonds breaks down in the high GSH environment of tumors to release drugs.	[84]
SSBPEI-DOX@ siRNAs/iRGD-PEG-HA	-	Ovarian cancer	A2780 cell	The nanoparticles significantly enhanced the antitumor effect of DOX compared with free DOX and also greatly suppressed the migration and invasion of A2780/DDP-derived CSCs.	[85]
scFv-P-BAP-siSurv-polyplex	Sense: 5′-GAAUUAACCCUU-GGUGAAU(tt)-3′	Prostate carcinoma	PC3 cell	Maltose-modified PPI significantly reduced cytotoxicity and improved biocompatibility. The single-chain antibody fragment (scFv) delivery system can specifically recognize prostate stem cell antigen (PSCA), thereby precisely entering target cells. The siRNA delivered by the delivery system significantly inhibited tumor growth.	[86]
H40-TEPA-PEG-MUC1 aptamer	Sense: 5′-GAAAGAAUUUGA-GGAAACUdTdT -3′ Antisense: 5′-AGUUUCCUC-AAAUUCUUUCdTdT 3′	Breast cancer	MCF-7 cell	It exhibits significant gene silencing effects in MCF-7 cells and demonstrates superior gene silencing efficiency compared to non-targeted dendritic polymers and Lipofectamine-2000.	[87]
Survivin siRNA/PTX PM	Sense: 5′-GCAUUCGUCCGG-UUGCGCUdTdT-3′	Breast cancer	SKOV3-tr cell MDA-MB 231 cell	Significantly inhibited cell viability and enhanced cell sensitivity to PTX in a variety of cancer cell lines, while effectively overcoming drug resistance by down-regulating survivin protein level and disrupting microtubule structure in drug-resistant cell line SKOV3-tr.	[88]
siRNA/PTX PM	Sense: 5′-GCAUUCGUCCGG-UUGCGCUdTdT-3′	Ovarian cancer	SKOV3-tr cell	PTX encapsulation efficiency to 90%, downregulated the expression of survivin, and promoted drug enrichment in tumor tissues.	[89]

**Table 3:** Targeted delivery of survivin siRNA by natural polymeric nanoparticles.

Natural Polymer-Based Survivin siRNA Delivery Systems					
Nanoparticle	Survivin Sequence	Cancer Types	Cell Lines	Outcome	Ref.
SUR siRNA-CS-g-PMMA/DS	Sense: 5′-GAACAUCAUCAU-CCAGGACTT-3′	Breast cancer	4T1 cell	4T1 cells treated with siSurvivin-loaded nanoparticles showed a significant decrease in cell viability, migration ability, and sphere size, confirming that this delivery system is effective in achieving gene silencing.	[100]
CE7Q/CQ/S/Survivin shRNA	Survivin-shRNA: 5′-GAATTA-ACCCTTGGTGAAT-3′	Lung cancer	A549 cell	This system could recognize epidermal growth factor receptor and enter into EGFR-mutated non-small cell lung cancer cells, and a stimuli-responsive release profile was achieved by near-infrared laser irradiation at pH 5.4 and displayed superior antitumor efficacy in vitro and in vivo.	[101]
PEG-CS/siRNA	-	Breast cancer	4T1 cell	PEG-CS/siSurvivin showed a significant inhibitory effect on 4T1 cells, while the cell survival rate decreased and the apoptosis rate elevated as compared to the naked siRNA group, inhibiting tumor growth and lung metastasis effectively.	[102]
TAT-g-CS/siSsur	Sense: 5′-GAACAUCAUCAU-CCAGGAC-3′	Breast cancer	MCF-7 4T1-Luc	Improved cellular uptake efficiency, inhibited the proliferation of 4T1-Luc tumor cells, and significantly inhibited the in vivo growth and metastasis of malignant breast tumors.	[103]
MT/PTX/pDNA/rhIL-2 NPs	-	Hepatocellular carcinoma	QGY-7703	Formation of MT nanoparticles with redox properties. The release rate significantly improved. Tumor inhibition rate was significantly higher than the treatment group using the three drugs alone, enhancing the antitumor effect.	[104]
PBA-COS/siRNA	Sense: 5′-GCAUUCGUCCGG-UUGCGCUTT-3′ Antisense: 5′-AGCGCAACCGGACGAAUGCTT-3′	Melanoma	B16F10 cell	Significantly inhibited melanoma cell proliferation, growth, and metastasis.	[105]
TS-MSN/siRNA	Sense: 5′-GUCUGGACCUCA-UGUUGUUdTdT-3′	Breast cancer	MDA-MB-231 cell	Improved cellular internalization and enhanced gene silencing efficiency.	[106]
TS-MSN siSurvivin	Sense: 5′-GUCUGGACCUCA-UGUUGUUdTdT-3′ Antisense: 5′-AACAACAUG-AGGUCCAGACdTdT-3′	Breast cancer	SK-BR-3 cell	TS-MSN improved cellular internalization and enhanced the anticancer effect of DOX by downregulating survivin expression.	[107]
CSP/TPE@siRNA-SP94	sense: 5′-CACCGCAUCUCU-ACAUUCATT-3′ Antisense: 5′-UGAAUGUAG-AGAUGCGGUGTT-3′	Hepatocellular carcinoma	Huh-7 cell	It exhibits excellent fluorescence and magnetic resonance imaging performance both in vitro and in vivo and significantly inhibits tumor growth in a nude mouse model carrying Huh-7 tumors.	[108]
DOX/siRNA/PEI-SFNPs	-	Breast cancer	4T1 cell	It effectively inhibited tumor growth without causing significant weight loss or other systemic toxicity, providing a new strategy for breast cancer treatment.	[109]
APR	Sense: 5′-GGACCACCGCAUCUCUACAdTdT-3′ Antisense: 3′-dTdTCCUGGU-GGCGUAGAGAUGU-5′	Breast cancer	MCF-7 cell	ErbB3 aptamer can specifically recognize and bind to the ErbB3 receptor overexpressed on the surface of breast cancer cells, realizing the precise delivery of siRNA. Protamine not only enhances the stability of the complex but also improves the intracellular delivery efficiency of siRNA through the electrostatic binding of siSurvivin.	[110]
NP-siRNA/Pt (IV)	Sense: 5′-GAAUUAACCCUU-GGUGAUTT-3′ Antisense: 3′-AUUCACCAAG GGUUAAUUCTT-5′	Lung cancer	A549 cell	Co-loading siSurvivin and Pt(IV) prodrug enhanced the tumor tissue-specific accumulation of the nanocarrier. Surface modification of the nanocarrier with PGA effectively improved its stability and prolonged its circulation time in the blood.	[111]
XL-DPs/siRNA	-	Lung cancer	A549 cell	The polymer is pH-responsive and releases siSurvivin in acidic environments. It has good biocompatibility and low cytotoxicity.	[112]
PEG–RAFF-siRNA	Sense: 5′-GAGACAGAAUAG-AGUGAUATT-3′ Antisense: 5′-UAUCACUCU-AUUCUGUCUCTT-3′	Breast cancer	MCF-7 cell	Arginine is frequently present in cell-penetrating peptides, which can enhance cellular uptake. Alanine serves as a spacer, which effectively avoids the steric hindrance.	[113]
VE-Sper/DSPE-PEG2000/siRNA	Sense: 5′-GCAUUCGUCCGG-UUGCGCUdTdT-3′ Antisense: 5′-AGCGCAACC-GGACGAAUGCdTdT-3′	Hepatocellular carcinoma	HepG2 cell	This nanocomposite can effectively silence the Eg5 and survivin genes and arrest the cell cycle at the G2/M phase. It significantly inhibits tumor growth in HepG2 tumor-bearing mice.	[114]

**Table 4:** Targeted delivery of survivin siRNA by liposomal polymer nanoparticles.

Liposomal Polymer-Based Survivin siRNA Delivery Systems					
Nanoparticle	Survivin Sequence	Cancer Types	Cell Lines	Outcome	Ref.
ALPR (HER2/CD44-Targeted Hydrogel Nanobot)	Sense: 5′-GCAUUCGUCCGG-UUGCGCUdtdt-3′ Antisense: 5′-AGCGCAACC-GGACGAAUGCdtdt-3′	Breast cancer	SKBR-3 MCF-7 MDA-MB-231	ALPR can efficiently deliver Herceptin, peptide, and survivin siRNA to SKBR-3, MDA-MB-231, and MCF-7 cells, demonstrating excellent antitumor effects.	[125]
cRGD-PSH-NP/S	Sense: 5′-mGCAGGUUCCUm-UAUCUGUCATT-3′	Hepatocellular carcinoma	HepG2 cell	cRGD-PSH-NP/S markedly downregulated survivin expression both in vitro and in vivo and exhibited potent tumor inhibition in HepG2-bearing nude mice.	[126]
Tf-L-SN38/P/siRNA	Sense: 5′-mGCAGGUUCCUm-UAUCUGUCAdTdT-3′ Antisense:5′-UGAmCAGAm-UAAGGAACCUGmCdTdT-3′	Cervical cancer	HeLa cell	Tf-modified liposomes can specifically bind to transferrin receptors on the surface of tumor cells, increasing drug accumulation in tumor tissues. This enhances the efficacy of chemotherapeutic drugs and inhibits tumor growth.	[127]
LNP/survivin siRNA	Sense: 5′-GGACCACCGCAUCUCUACATT-3′ Antisense: 5′-UGUAGAGAU-GCGGUGGUCCTT-3′	Human retinoblastoma cell line	Y79 cell	Downregulation of survivin via siLNP enhances the cytotoxic effects of carboplatin and melphalan on Y79 cells and primary retinoblastoma cells.	[128]
HA/HAase/CS/liposome/survivin-shRNA (HCLR)	shRNA: 5′-AATTTGAGGA-AACTGCGGAGA-3′	Breast cancer	MDA-MB-231 cell	HA specifically binds to the CD44 receptor on the surface of tumor cells, promoting tumor targeting and prolonging the retention time of liposomes in the bloodstream. CS undergoes protonation in the tumor microenvironment (pH 6.5) to promote cellular uptake, while pH-triggered HAase release improves the diffusion of the nanocarrier in tumor tissue, enhancing tumor penetration. It exhibits excellent in vivo tumor suppression capabilities.	[130]
siRNA-LAPA_LBNP	Sense: 5′-GUCUGGACCUCA-UGUUGUUdTdT-3′	Breast cancer	SKBR-3 cell	It has good physical and chemical properties, a high cell uptake rate, and significant cytotoxic effects.	[131]
OCA-CC-siRNA	Sense: 5′-GAAUUUGAGGAA-ACUGCGAtt-3′ Antisense: 3′-ttCUUAAACU-CCUUUGACGCU-5′	Breast cancer	MCF-7	Enhanced cytoplasmic delivery capacity and demonstrated significant antitumor activity in vitro and in vivo through silencing survivin expression.	[132]

**Table 5:** Targeted delivery of survivin siRNA by organic/inorganic hybrid nanomaterials.

Organic/Inorganic Hybrid Nanomaterials-Based Survivin siRNA Delivery Systems					
Nanoparticle	Survivin Sequence	Cancer Types	Cell Lines	Outcome	Ref.
CaP-NPs-siRNA	Sense: 5′-CUAUUGUGACCU-GGACUUATT-3′ Antisense: 5′-UAAGUCCAG-GUCACAAUAGAG-3′	Glioblastoma	F98 cells	o14PEGMA improves the dispersion stability of nanoparticles, increases cellular uptake efficiency, and reduces the potential cytotoxicity of traditional CaP. Calcium phosphate nanoparticles protect siRNA from degradation and promote its uptake into cells.	[134]
CaP-Arg-siRNA	Sense: 5′-GAAGCAGUUUGA-AGAAUUATT-3′ Antisense: 5′-UAAUUCUUC-AAACUGCUUCTT-3′	Lung cancer	A549 cell	CaP-Arg-siRNAs significantly suppressed the expression of survivin and cyclin B1, resulting in a marked decrease in cell growth and inducing apoptosis drastically.	[135]
NDCONH(CH2)2NH-VDGR/survivin siRNA	Sense: 5′-GCATGGGTCCCC-CGACGTTG-3′ Antisense: 5′-GCTCCGGCC-AGAGGCCTCAA-3′	Breast cancer	MCF-7 cell	Reduced survivin expression and displayed a potent inhibitory effect on tumor growth in vitro and in vivo.	[136]
CNTs-PS/siRNA	Sense: 5′-CACCGCAUCUCU-ACAUUCATT-3′ Antisense: 5′-UGAAUGUAG-AGAUGCGGUGTT-3′	Cervical cancer	HeLa cell	The modified CNTs demonstrated excellent biocompatibility and temperature-sensitive properties. The combination of photothermal therapy and gene therapy caused massive apoptosis in HeLa cells and exhibited strong antitumor activity in vivo.	[139]
CMOL@ siRNA	SUR A: Sense: 5′-CCUUCCUCACUG-UCAAGAATT-3′ Antisense: 5′-UUCUUGACA-GUGAGGAAGGTT-3′ SUR B: Sense: 5′-GAGACCAACAAC-AAGCAAATT-3′ Antisense: 5′-UUUGCUUGU-UGUUGGUCUCTT-3′ SUR C: Sense: 5′-CUACCCGUCAGU-CAAUUGATT-3′ Antisense: 5′-UCAAUUGAC-UGACGGGUAGTT-3′	Colorectal and Breast cancer	CT26, 4T1 cell	CMOL functions as an effective reactive oxygen species (ROS) generator, and the cationic framework serves as an siRNA cocktail carrier to synergistically downregulate survivin levels. Combined with low-dose X-ray irradiation, it has shown significant antitumor efficacy.	[140]
PEG-CPP33@ORI@survivin siRNA@ZIF-90	Antisense: 5′-UAAUUCUUC-AAACUGCUUCTT-3′	Lung cancer	A549 cell	PEG-CPP33@NPs significantly improved the cellular uptake efficiency of siRNA in A549 cells and showed stronger tumor accumulation. The strongest antitumor effect in vivo was observed through the co-delivery of ORI and survivin siRNA.	[141]
siRNA@MOF-GalNAc	Sense: 5′-AAGGAGAUCAAC-AUUUUCA-3′ Antisense: 5′-UGAAAAUGU-UGAUCUCCUU-3′	Hepatocellular carcinoma	HepG2 cell	The GalNAc-decorated MOF specifically accumulated in HCC tumor tissue and was effectively endocytosed by HCC cells. The protective properties of the MOF increased the stability of siRNA and allowed for significant downregulation of survivin expression in HCC tumors, contributing to tumor inhibition through the suppression of cell proliferation and the induction of apoptosis.	[142]

## Data Availability

No new data were created or analyzed in this study.

## Abbreviations

AICAR	5-Aminoimidazole-4-carboxamide ribonucleotide
AIE	Aggregation-induced emission
AIF	Apoptosis-inducing factor
ALPR	HER2/CD44-targeted hydrogel nanobot
ASOs	Antisense oligonucleotides
CA	Cationized amylose
cIAP1	Cellular inhibitor of apoptosis protein 1
cIAP2	Cellular inhibitor of apoptosis protein 2
COS	Chitooligosaccharides
CPC	Chromosal passenger complex
CPP	Cell penetrating peptide
DDP	Cis-Diaminodichloroplatinum/Cisplatin
DIABLO	Direct inhibitor of apoptosis-binding proteins with low pI
DLPC	1,2-dilauroyl-.sn-glycero-3-phosphocholine
DOX	Doxorubicin
DPPC	1,2-dipalmitoylsn-glycero-3-phosphocholine
DSPE	1,2-distearoyl-sn-glycero-3-phosphoethanolamine
ECVs	Extra cellular vesicles
HA	Hyaluronic acid
HCC	Hepatocellular carcinoma
IAP	Inhibitor of apoptosis proteins
INCENP	Inner centromere protein
LA	Linolic acid
LNCs	Lipid nanocapsules
LNPs	Liquid nanoparticles
LPHNPs	Lipid polymer hybrid nanoparticles
MA	Mercaptobenzoic acid
ML-IAP	Melanoma inhibitor of apoptosis protein
MTS	Mitochondrial targeting sequence
NAIP	Neuronal apoptosis inhibitory protein
NES	Nuclear export signal
OA	Oleic acid
ORI	Oridonin
PA	Palmitic acid
PAMAM	Polyamide-amine
PBA	Phenylboronic acid
PC	Phosphatidyl choline
PCL	Polycaprolactone
PEG-CS	Poly (ethylene glycol)-modified chitosan
PEG-PAsp	Poly(ethylene glycol)-poly (aspartic acid)
PEI	Polyethyleneimine
PEI–PLA	Polyehtyleneimine-poly(lactic acid)
PGA	Polyglutamic acid
PM	Polymeric micelles
PNPs	Polymer nanoparticles
PSCA	Prostate stem cell antigens
PSH	PEI–SS–HA
PTT	Photothermal therapy
PTX	Pachitaxel
RNAi	RNA interference
ROS	Reactive oxygen species
SAC	Spindle assembly check point
SFNPs	Silk fibroin nanoparticles
siRNA	Small interfering RNA
SMAC	Second mitochondria derived activator of caspases
SPC	Soya phosphatidylcholine
SPIO	Superparamagnetic iron oxide
Survivin	BIRC5
TEPA	Tetraethylenepentamine
TKI	Tyrosine kinase inhibitor
TMC	N, N, N-trimethyl chitosan
TPE	Tetraphenylethylene
XIAP	X-linked inhibitor of apoptosis protein
